# Vanishing retinal arterial aneurysms with anti-tubercular treatment in a patient presenting with idiopathic retinal vasculitis, aneurysms, and neuroretinitis

**DOI:** 10.1186/s12348-016-0074-3

**Published:** 2016-02-27

**Authors:** Ramandeep Singh, Kusum Sharma, Aniruddha Agarwal, Mohit Dogra, Vishali Gupta, Aman Sharma, Mangat R. Dogra

**Affiliations:** Advanced Eye Center, Postgraduate Institute of Medical Education and Research, Sector 12, Chandigarh, 160012 India; Department of Medical Microbiology, Postgraduate Institute of Medical Education and Research, Chandigarh, India; Stanley M. Truhlsen Eye Institute, University of Nebraska Medical Center, Omaha, NE USA; Department of Internal Medicine, Postgraduate Institute of Medical Education and Research, Chandigarh, India

**Keywords:** IRVAN, Intraocular tuberculosis, Anti-tubercular therapy, Aneurysms, Fluorescein angiography, Uveitis, Polymerase chain reaction

## Abstract

**Background:**

Idiopathic retinal vasculitis, aneurysms, and neuroretinitis (IRVAN) syndrome presents with characteristic clinical manifestations such as aneurysms at arteriolar bifurcations and optic nerve and retinal vascular inflammation. Regression of such features on treatment with anti-tubercular therapy (ATT) combined with corticosteroids has not been reported in literature.

**Findings:**

A 30-year-old female with sudden painless decreased vision in the left eye was referred with a diagnosis of presumed tuberculous retinal vasculitis and a positive tuberculin skin test. Based on the clinical and angiographic features of the right eye, a diagnosis of IRVAN syndrome was made. In the left eye, the patient had vitreous hemorrhage for which pars plana vitrectomy was performed. The vitreous sample was positive for *Mycobacterium tuberculosis* using multiplex polymerase chain reaction, and the patient was started on standard four-drug ATT and oral corticosteroids. At 6-month follow-up, vanishing of retinal arterial aneurysms was observed.

**Conclusions:**

The pathogenesis of IRVAN syndrome is uncertain. One of the postulates is that the features of arterial aneurysms and other retinal vascular alterations occur secondary to acquired inflammatory reaction. We hypothesize that IRVAN syndrome may be a morphological diagnosis possibly associated with various entities, one of which could be ocular tuberculosis. It may be prudent to rule out intraocular tuberculosis in cases labeled as IRVAN syndrome in an endemic population.

## Findings

### Introduction

Idiopathic retinal vasculitis, aneurysms, and neuroretinitis (IRVAN) syndrome is a well-defined disease entity, which is diagnosed on the basis of constellation of clinical features such as retinal vasculitis, aneurysms at arteriolar bifurcations, and neuroretinitis (major criteria). In addition, patients with IRVAN syndrome may have a presence of retinal peripheral capillary non-perfusion, retinal neovascularization, and other secondary vasoproliferative complications, and macular exudation (minor criteria) [1, 2].

Earlier regarded as a benign self-limiting entity, recent studies have suggested that patients with IRVAN syndrome may continue to lose vision if left untreated. Various therapeutic regimens have been advocated for the treatment of IRVAN syndrome, including pan-retinal laser photocoagulation (PRP), surgery, trans-scleral cryotherapy, corticosteroid therapy, administration of monoclonal antibodies such as ranibizumab, and immunomodulatory therapy [2–4].

While a number of studies have suggested a diverse range of treatment options for this condition, the exact pathogenesis of IRVAN syndrome still remains unknown. Associations of IRVAN syndrome with allergic fungal sinusitis [5], elevated intracranial pressure [6], hyperhomocysteinemia [7], and p-ANCA [8] have been reported in literature. In addition, similarities in the pathogenesis of IRVAN syndrome and presumed tuberculous retinal vasculitis have been suggested [9]. We hereby report a case diagnosed as IRVAN syndrome based on clinical and imaging features, in which the retinal aneurysms vanished on treatment with anti-tubercular therapy (ATT).

### Case report

A 30-year-old female presented with sudden painless decreased vision in the left eye (OS) for the past 1 week. She was diagnosed elsewhere with presumed tuberculous retinal vasculitis with positive tuberculin skin test and was referred to the Retina Clinic, Postgraduate Institute of Medical Education and Research (PGIMER), Chandigarh, India, for further management. Her best-corrected visual acuity (BCVA) was 20/40 in the right eye (OD) and counting fingers close to the face in OS. The pupillary reactions were normal in both the eyes (OU). Intraocular pressure was 16 mmHg OU on Goldmann applanation tonometry. Anterior segment examination on slit-lamp biomicroscopy was unremarkable OU.

**Fig. 1:**
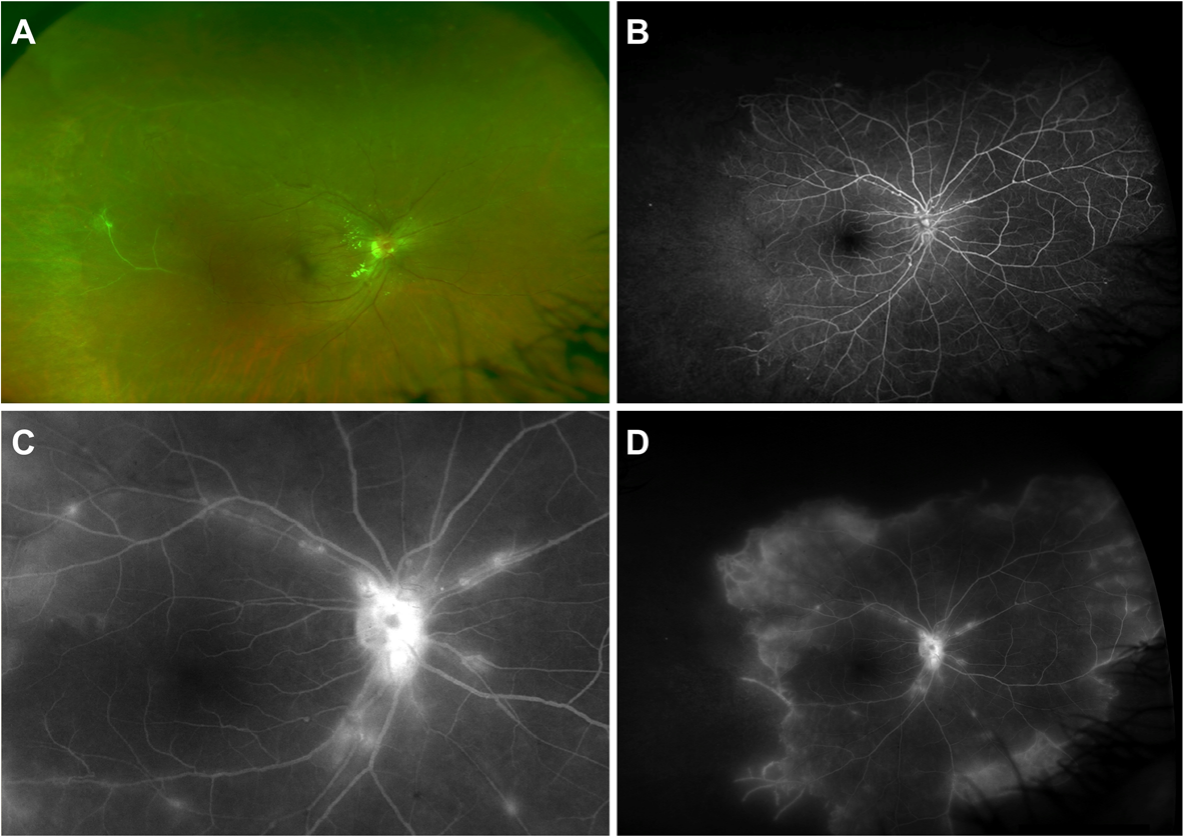
Fundus photograph (**a**) and fluorescein angiogram (FA) (**b**–**d**) of the right eye at presentation showing hard exudates, superficial hemorrhages, and “tied-knot-like” aneurysmal dilatations of the arterioles on optic disc and in the surrounding region. In addition, vascular sheathing can be observed in the larger vessels temporally. A large area of capillary non-perfusion can be seen temporally on FA (**b**). In the late phase (**d**), significant leakage can be observed from the peripheral vessels at the junction of perfused and non-perfused retina

Posterior segment examination showed hard exudates in the posterior pole and few superficial hemorrhages associated with “tied-knot-like” aneurysmal dilations of the arterioles on the optic nerve head and in the surrounding peripapillary area in OD (Fig. 1a). Fluorescein angiogram (FA) confirmed the presence of multiple aneurysms along the retinal arterioles (Fig. 1b–d). There was significant peripheral retinal non-perfusion in all the four quadrants OU (Fig. 1d, only the right eye shown). There was a presence of vitreous hemorrhage, peripapillary fibrovascular proliferation, sheathed vessels, and a featureless, ischemic retina in OS. In addition, there were multiple occluded small vessels in the periphery in OU (Fig. 1a). There was no evidence of macular edema on optical coherence tomography. Based on the clinical and FA features, diagnosis of IRVAN syndrome stage 2 in OD and stage 3 in OS was made [10].

Recording of thorough medical history and systemic examination to rule out ocular/systemic vasculitis and connective tissue disorders was performed but was unrevealing. There was no positive family history. The systolic and diastolic blood pressures were 116 and 76 mmHg, respectively, and there were no echocardiographic abnormalities. Laboratory investigations including chest x-ray and x-ray of the paranasal sinuses (water’s view), complete blood count, erythrocyte sedimentation rate, routine blood chemistry, complete lipid profile, urine analysis, carotid Doppler, Treponema pallidum hemagglutination test, anti-nuclear antibody, anti-double-stranded DNA antibody, anti-phospholipid antibodies (including anti-cardiolipin and lupus anticoagulant), serum homocysteine, anti-neutrophil cytoplasmic antibody, and positron emission tomography were negative. The tuberculin skin test (50 × 51 mm) and erythrocyte sedimentation rate (40 mm at the end of 1 h) were positive. High-resolution contrast-enhanced chest scan showed calcification in the left lower lobe suggestive of Ghon complex.

**Fig. 2:**
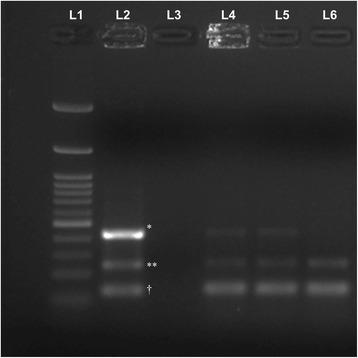
Positive results of multiplex polymerase chain reaction demonstrating positivity to tubercular antigens in the vitreous fluid. Band *L1* represents 100-bp molecular marker; *L2* is the positive control (*asterisk* indicates protein B (419 bp), *double asterisks* indicate MPB64 (240 bp), *dagger* indicates IS6110 (123 bp)). Band *L3* is the negative control. Bands *L4*, *L5*, and *L6* represent the positive results obtained from the vitreous fluid of the patient described in the index case

**Fig. 3:**
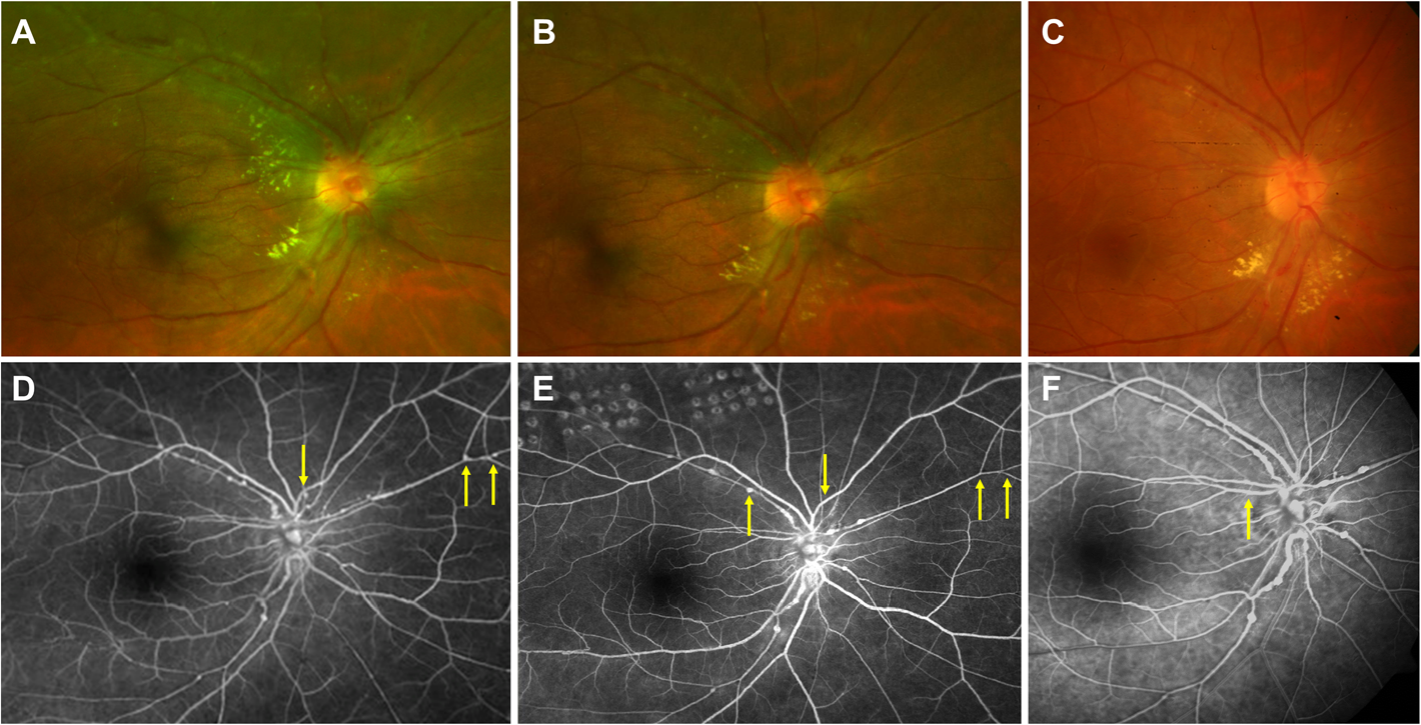
Comparison of the posterior pole fundus photographs at baseline (**a**), 6 months (**b**), and 11 months (**c**) of the right eye posterior pole (*magnified*) shows resolution of arterial aneurysms with resolution of hard exudates. Fluorescein angiogram (FA) at baseline (**d**) versus 6 month (**e**) and 11 month (**f**) shows much reduced aneurysmal dilations at arteriolar branching (*yellow arrows*). Laser scars of pan-retinal photocoagulation are clearly demonstrated in the FA at month 6 superiorly (**e**)

Pan-retinal photocoagulation was performed in the area of avascular peripheral retina in OD. The left eye underwent pars plana vitrectomy (PPV) and endolaser in the areas of non-perfusion. A vitreous sample was obtained at the time of surgery and was preserved for further analysis. Multiplex polymerase chain reaction (MPCR) was performed on the vitreous sample using a previously described technique [11]. The sample was positive for *Mycobacterium tuberculosis* (Fig. 2). The diagnosis was revised, and the patient was started on a tapering dose of oral prednisolone (1 mg/kg) for 3 months along with standard four-drug anti-tubercular treatment (ATT). At 6-month follow-up, BCVA improved to 20/20 in OD and 20/40 in OS. Posterior segment examination and FA imaging showed resolution of retinal arterial aneurysms in OU (Fig. 3; only the right eye shown). At the last follow-up visit, 11 months from the initial presentation, the patient had a BCVA of 20/20 in both the eyes. The retinal arterial aneurysms further decreased in number (Fig. 3). The anti-tubercular therapy is planned to be discontinued after completion of 1 year.

### Discussion

The natural history and pathogenesis of IRVAN syndrome has not been clearly elucidated yet. It has been postulated that the features of arteriolar aneurysms and other retinal vascular alterations such as occlusion occur as a result of pathological changes triggered by an acquired inflammatory reaction [9]. Sashihara et al. [12] have proposed that the presence of anterior chamber and vitreous cells in certain patients with IRVAN syndrome and changes in the morphological appearance and location of the aneurysms could be the result of a migratory inflammatory process involving alternate segments of the retinal vascular network.

Among patients with IRVAN syndrome, this inflammatory process may be activated by hypersensitivity reaction to tubercular antigens [9]. It has been speculated that the pathogenesis of IRVAN syndrome may be related to presumed tuberculous retinal vasculitis since the findings in IRVAN syndrome resemble those of acquired allergic vascular diseases [9]. Peripheral retinal non-perfusion may occur in both the entities except for aneurysms at arteriolar bifurcations, which are a characteristic feature of IRVAN syndrome. A number of studies have demonstrated a strong association between retinal vasculitis and tuberculosis [13–16]. Thus, IRVAN syndrome can be considered to be a morphological diagnosis such as serpiginous choroidopathy, which may have various associations including ocular tuberculosis.

IRVAN was initially thought to be a self-limiting disease. Chang et al. [1] in 1995 reported a more aggressive course with devastating visual consequences. Samuel et al. [10] presented the largest cohort of patients with IRVAN syndrome and recommended early pan-retinal laser photocoagulation. All eyes with vitreous hemorrhage underwent early PPV, and extensive pan-retinal photocoagulation was done in the fellow eye [10]. The role of anti-inflammatory drugs, i.e., steroids, and immunosuppressive drugs is still unclear [1, 2]. In the index case, treatment with ATT and corticosteroids (for the initial 3 months) may have controlled the underlying inflammatory/hypersensitivity stimulus resulting in resolution of arteriolar aneurysms.

Regression of arteriolar aneurysms among patients with IRVAN syndrome is extremely rare. There are very few case reports that have demonstrated resolution of aneurysms in the literature [5, 12, 17]. In one of these reports by Owens and Gregor [17], the arteriolar aneurysms vanished over 7 years after the patient underwent surgery for vitreous hemorrhage. Pan-retinal photocoagulation may also be associated with regression of retinal aneurysms in IRVAN [10]. In addition, adjunctive corticosteroid therapy in our patient may have also played a role in resolution of aneurysms.

Despite the limitation of being a single case, it is imperative to note that our patient had a strongly positive laboratory assay for tubercular antigens and vitreous fluid positive for mycobacterium genome using the PCR technique. While it is possible that the laboratory findings of ocular tuberculosis were coincidental, PCR has been reported to have a high specificity and positive predictive value (100 %) [11].

### Conclusions

Based on the findings of the index case, we hypothesize that the morphological features of IRVAN syndrome may occur as an extended spectrum of ocular tuberculosis in endemic regions with high prevalence of the disease. In conclusion, the index case suggests that intraocular tuberculosis can be associated with typical features of IRVAN and must be investigated in endemic population.

### Consent

Written informed consent was obtained from the patient for the publication of this report and any accompanying images.
